# Effectiveness of skull X-RAY to determine cochlear implant insertion depth

**DOI:** 10.1186/s40463-018-0304-9

**Published:** 2018-09-03

**Authors:** Vinay Fernandes, Yiqiao Wang, Robert Yeung, Sean Symons, Vincent Lin

**Affiliations:** 1 Division of Otolaryngology – Head and Neck Surgery, Faculty of Medicine, University of Toronto, Toronto, Canada; 2 Faculty of Medicine, University of Ottawa, Ottawa, Canada; 3 Division of Radiology, Faculty of Medicine, University of Toronto, Toronto, Canada

**Keywords:** Cochlear implant, Insertion depth, CT, X-RAY

## Abstract

**Background:**

Cochlear implant (CI) insertion depth can affect residual hearing preservation, tonotopic range coverage, and Mapping. Therefore, determining insertion depth has the potential to maximize CI performance. A post-op skull X-RAY is commonly used to assess insertion depth, however its effectiveness has not been well established. Our primary objective was to assess the accuracy of post-op skull X-RAYs to determine insertion depth, compared to CT as the gold standard. Secondary objectives were to compare experience level of raters and different skull X-RAY views.

**Methods:**

Thirteen patients with Advanced Bionic HiRes 90 K implants, and post-operative temporal bone CT scans were selected from the CI database at Sunnybrook Health Sciences Centre. Medical students, otology fellows, and CI surgeons evaluated insertion depths on post-op skull X-RAYs, while neuroradiologists evaluated CT scans. Descriptive statistics, regression analysis, and paired t-tests were used to compare the two types of imaging.

**Results:**

X-RAYs and CTs provided an equivalent mean insertion depth of 337 degrees (*p* = 0.93), a mean difference of − 0.9 degrees and a standard deviation of paired differences of 43 degrees. Although means were similar across rater groups, CI surgeons (45 degrees) had the lowest standard deviation of paired differences. Comparing X-RAY views, Caldwell (29 degrees) had less variation than Towne (59 degrees) for standard deviation of paired differences.

**Conclusions:**

Skull X-RAYs provide accurate and reliable measurements for CI insertion depth. The Caldwell view alone may be sufficient for evaluations of insertion depth, and experience has a minor impact on the variability of estimates.

**Electronic supplementary material:**

The online version of this article (10.1186/s40463-018-0304-9) contains supplementary material, which is available to authorized users.

## Background

The cochlear implant (CI) converts acoustic energy into electrical stimuli, bypassing hair cells to directly stimulate spiral ganglion neurons using a series of platinum electrodes [1]. CIs with longer electrode arrays that are inserted deeper and closer to the apex of the cochlea can potentially increase tonal range [2]. With greater insertion depth, hearing perception, including Hearing In Noise and Consonant Nucleus Consonant test scores appear to improve in some studies [3–5]. Conversely, deeper insertion can also increase iatrogenic injury to the cochlea, leading to decreased hearing preservation [6]. Although “soft surgery” techniques involving use of corticosteroids, and scala tympani insertion can minimize this damage, shallower insertion depths are still associated with a lower rate of iatrogenic injury [6–9]. Previously it was believed that CI surgery would destroy all residual hearing. It is now accepted that hearing preservation is possible, and should be maximized [6, 10].

Imaging post-operatively to determine CI electrode insertion depth and placement may vary greatly and include Computerized Topography (CT) or skull X-RAY [11–13]. CT is a highly accurate technique physicians currently utilize and can be reconstructed to yield 3D high-resolution data to determine electrode position [12, 14–16]. More commonly, immediate post-operative skull X-RAYs are performed following CI surgery at implant centres. At Sunnybrook Health Sciences Centre (SHSC), our patients undergo a routine three-view series of X-RAYs often the night of their surgery, or occasionally the day afterwards. These X-RAYs help confirm appropriate electrode placement within the cochlea including insertion depth, as well as identify kinking, squeeze, and integrity of the electrode [12].

Although skull X-RAYs are commonly used in practice for CIs, and many studies have used X-RAYs to determine insertion depth, accuracy of skull X-RAYs have not been fully established. Furthermore, few studies have compared skull X-RAYs to other modalities for insertion depth estimates [13, 17]. Our primary objective was to evaluate the accuracy of post-op skull X-RAYs to determine insertion depth when compared to CT. Comparison of insertion depths by experience level, and by different X-RAY views were assessed in a secondary analysis.

## Methods

### Study population

Institutional Research Ethics Board approval was obtained. Patients from 2003 to 2009 were selected from an existing database of CIs at SHSC. We included adults (≥18 years old) who had a post-operative CT scan of their temporal bones. CT scans were only provided for the unique circumstance of being considered for a second contralateral CI. Only Advanced Bionic HiRes 90 K implants were included; this eliminated the potential variable of more than one type of electrode being studied [18]. Patients were excluded if they already had other types of CIs, or were missing a postoperative skull Caldwell or Towne view in their X-RAY series. The Stenvers view was not available for most patients and was therefore not assessed.

### Skull X-RAY imaging

Skull X-RAYs were obtained from the SHSC system. All skull X-RAYs were performed within 24 h of surgery. The Caldwell and Towne views were optimized in the radiology suite by the principle investigator to provide best face value images of the implants. De-identified images were presented on PowerPoint (Microsoft ©) and all images were randomized using a random number generator. Rater participants included medical students, otology fellows, and CI surgeons. All groups were instructed to estimate CI insertion depth in degrees based on post-op skull X-RAYs. We used the round window as the zero degree standard [16].

### CT imaging

Two experienced neuroradiologists at our institution interpreted the actual degree of insertion using CTs. CT images of temporal bones were reviewed in sequence. Degree of insertion was rated on a 360° scale. Neuroradiologists were blinded to previous CT reports, and patient IDs were removed to eliminate detection bias.

### Statistical analysis

Statistical analysis was performed using SPSS software ©. Inter-rater reliability between neuroradiologists as well as between-rater groups was determined using intra-class correlations (ICC). Skull X-RAY insertion depth estimates were compared to CT using descriptive statistics, regression analysis, and paired t-tests. A *p*-value of *<* 0.05 was used for statistical significance.

## Results

### Demographics

In total, we selected 13 patients who underwent CT scans of their temporal bones following cochlear implantation. These patients had 21 skull X-RAYs, including 12 Caldwell views and 9 Towne views available for comparison. Two CI surgeons, four otology fellows, and seven medical students participated in skull X-RAY insertion depth estimations, providing a total of 273 X-RAY estimates.

### Comparison of skull X-RAY to CT

**Fig. 1:**
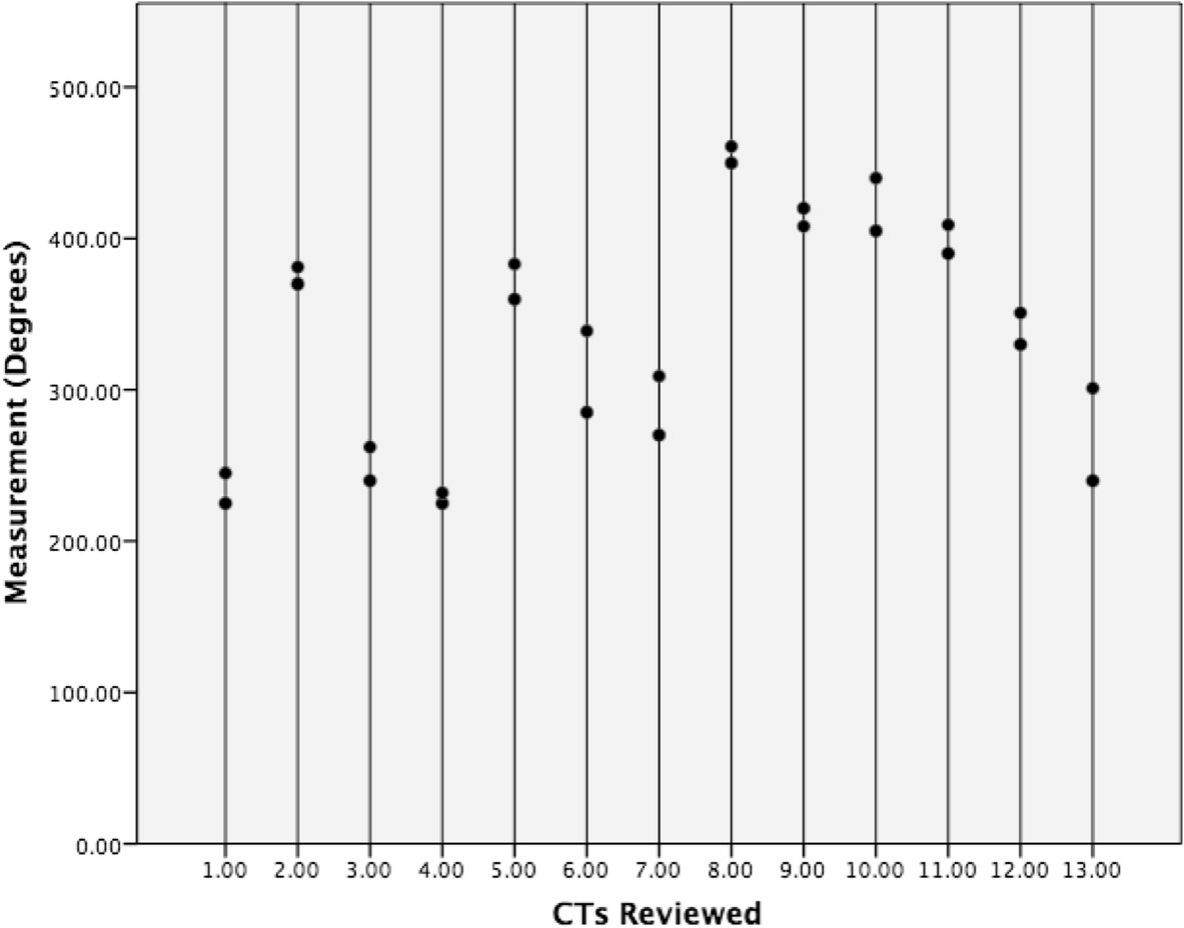
Dot plot of neuroradiologist agreement. Intra-Class Coefficient = 0.962, 95% confidence interval is between 0.549 and 0.990

**Fig. 2:**
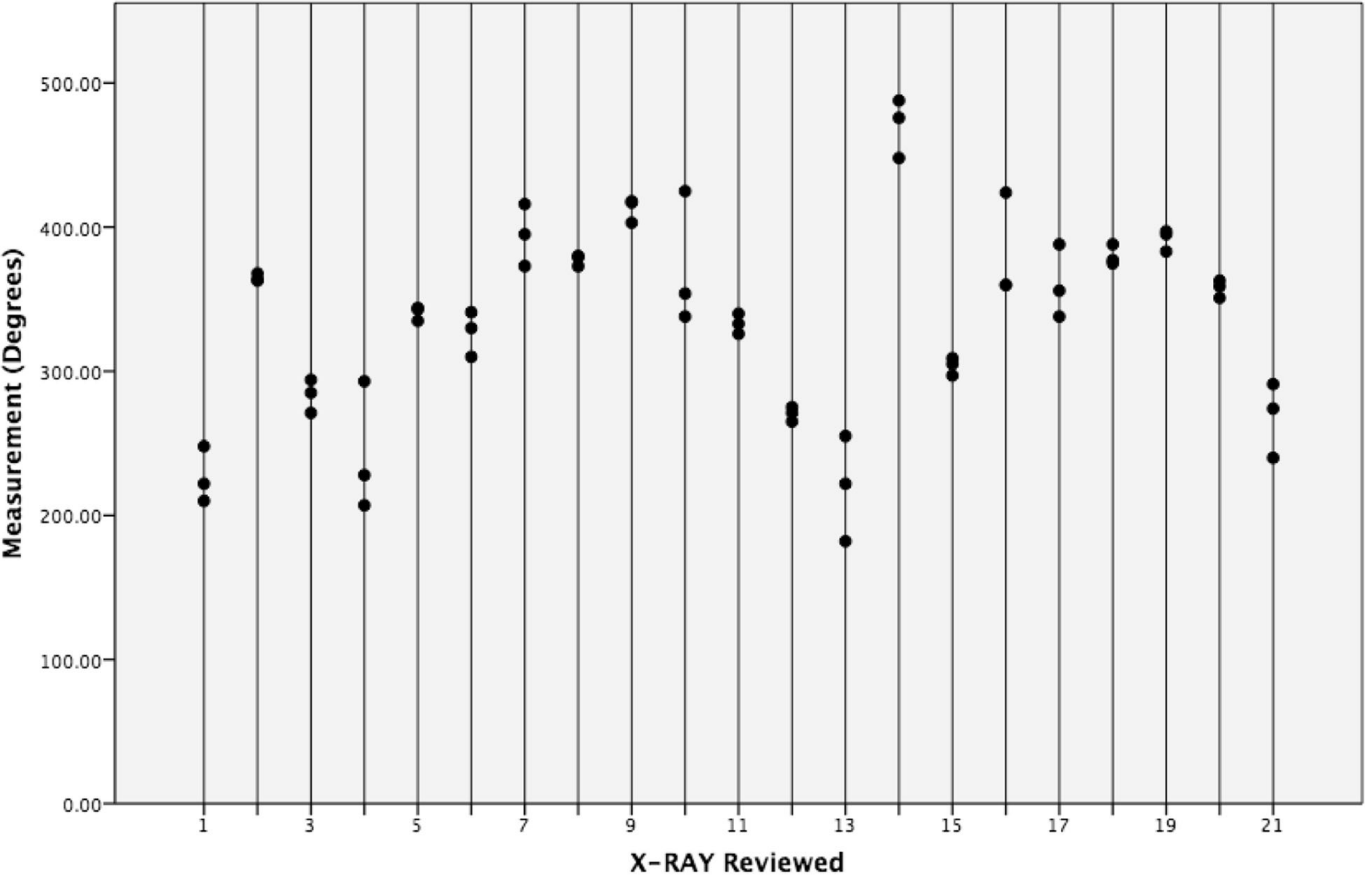
Dot plot of rater agreement. Intra-Class Coefficient = 0.963, 95% confidence interval is between 0.476 and 0.992

**Fig. 3:**
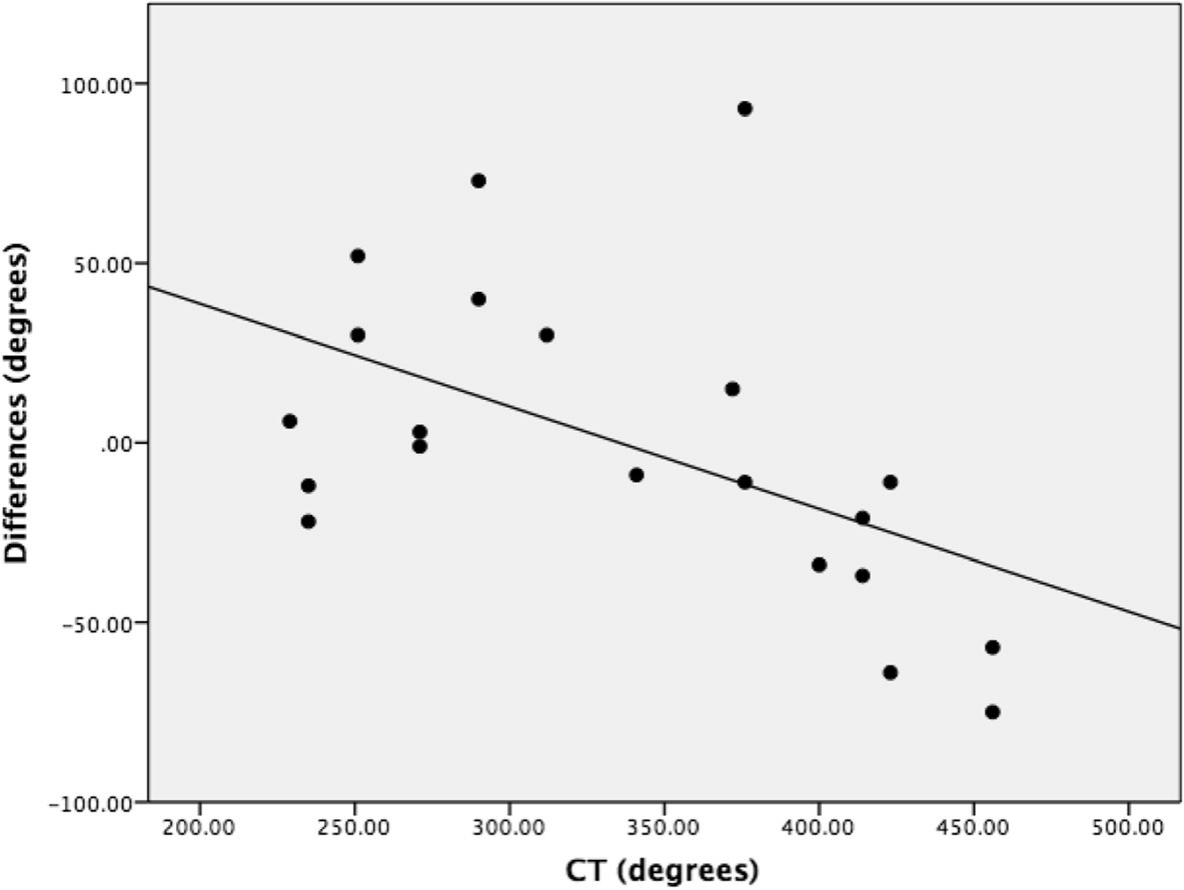
Differences between X-RAY and CT insertion depths. Absolute differences increase as insertion depth deviates from 360 degrees

ICC Coefficients showed a high reliability of estimates between neuroradiologists (ICC = 0.962, Fig. 1) and between raters (ICC = 0.963, Fig. 2). Mean X-RAY insertion depth estimates were equivalent to mean CT insertion depth estimates at 337 degrees. Mean difference was − 0.9° (95% confidence interval − 20.6 to 18.8) and the standard deviation of paired differences was 43° (Tables 1 and 2). The estimated X-RAY insertion depths correlate with CT without significant differences between estimations (*p =* 0.93). The linear regression was inversely proportional, representing an underestimation on skull X-RAY for insertions higher than 360°, and an overestimation for insertions lower than 360° (Fig. 3).

**Table 1:** Group means and standard deviations

Group	Mean (degrees)	Standard deviation (degrees)
CI surgeons	335	65
Fellows	341	63
Medical students	335	76
All raters	337	67
Caldwell	323	70
Towne	351	86
CT	337	79

**Table 2:** X-RAY versus CT insertion depth estimates

Group	Standard deviation of paired differences (degrees)	Mean difference (degrees)	Maximum difference (degrees)	Minimum difference (degrees)	*P*-value
CI surgeons	45	−2.0	99	−68	0.84
Fellows	49	3.2	112	−83	0.76
Medical students	47	−2.2	100	−78	0.83
All raters	43	−0.9	93	−75	0.93
Caldwell	29	−5.8	40	−57	0.51
Towne	59	5.7	93	−75	0.78

### Impact of rater experience

**Fig. 4:**
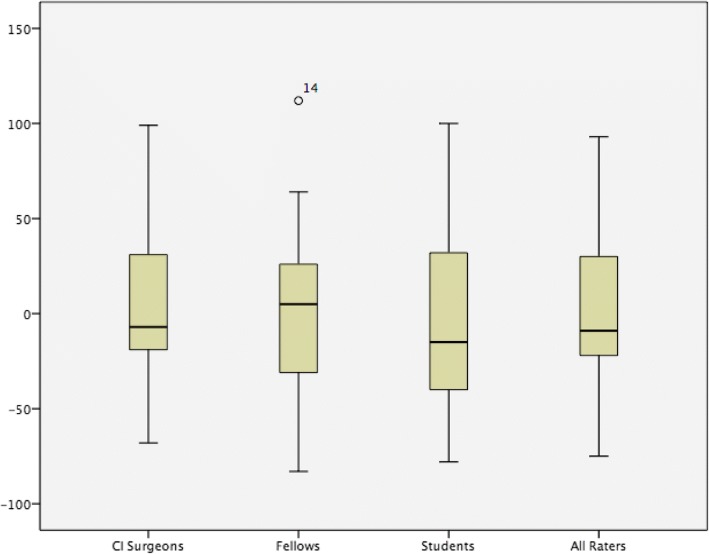
Box plot of paired differences by experience level. Mean differences of all groups approach zero

The three rater groups, medical students, otology fellows, and CI surgeons, were analyzed individually for the correlation of their skull X-RAY estimates to CTs. Estimations from the seven medical students resulted in a mean of 335° (*p* = 0.83), a mean difference of − 2.2° and standard deviation of paired differences of 47° (Tables 1 and 2). Estimations from the four fellows resulted in a mean of 341° (*p* = 0.76), a mean difference of 3.2°, and a standard deviation of paired differences of 49° (Tables 1 and 2). Similarly, the six CI surgeons had a mean of 335° (*p* = 0.84), a mean difference of − 2.0 degrees, and a standard deviation of paired differences of 45° (Tables 1 and 2). Paired differences of the rater groups were represented on box plots (Fig. 4).

### Comparison of skull X-RAY views

Rater estimates for the two skull X-RAY views of Caldwell and Towne were compared. The mean estimate for the Caldwell view was 323°, while the paired mean CT estimate was 333° (*p* = 0.51). Likewise, the mean estimate for the Towne view was 351 degrees, while the paired mean CT estimate was 343 degrees (*p* = 0.78, Table 1). Compared to CT, the Caldwell view appears to underestimate while the Towne view appears to overestimate. Although mean differences are similar, there was a sizeable difference when comparing standard deviation of paired differences (Table 2). Caldwell (29 degrees) provided a more precise estimate than Towne view (59°) (Table 3).

**Table 3:** Standard deviation of paired differences (Degrees)

X-RAY view	Surgeons	Fellows	Students	All raters
Caldwell	33	38	30	29
Towne	58	63	64	58

## Discussion

CI insertion depth has become an important metric to quantify in cochlear implant patients. Accurate determination may potentially impact on implant performance including mapping parameters. However, few studies have discussed the effectiveness of X-RAYs post-operatively. One study by Syrakic et al., demonstrated the strength of the radiograph to estimate the angular depth of insertion, and used CT scans to assess error on X-RAY. Our study adds support to their findings for the efficacy of skull X-RAYs, shows validity at a Canadian centre, and evaluates differences by rater and skull X-RAY views [13, 17].

Mean differences showed skull X-RAY estimates to be very similar to CT, and no statistical significant differences were found. Given that we used a 360-degree scale to rate insertion depth, the standard deviation of paired differences of 43 degrees between the two types of imaging supports a low variability of skull X-RAY estimations. Our skull X-RAY estimates were less accurate as CI insertion depths move further from 360 degrees. Therefore, skull X-RAYs may not be as useful for insertion depths that are extremely deep or shallow. This may become problematic, as the extremes in insertion depth likely have the greatest impact on CI performance.

**Fig. 5:**
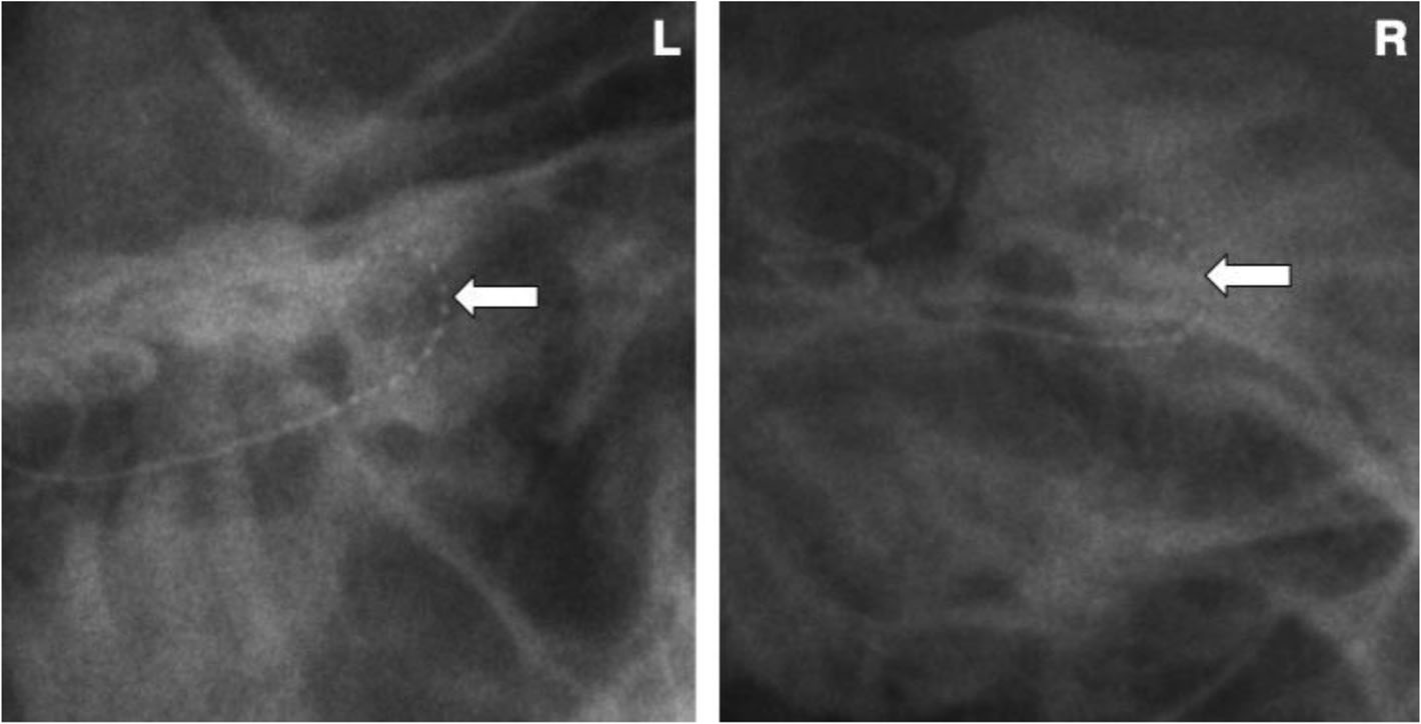
Left Caldwell and right Towne views. Arrows are pointing to electrode position. Notice an easier visualization of the electrode spiral on the Caldwell view

At our centre, Caldwell and Towne skull X-RAY views are commonly used for patients post CI surgery. Our study suggests the Caldwell view is less variable than the Towne view for estimating electrode insertion depth, and therefore the Towne view may not be required post-op. Caldwell is a less angled anteroposterior view, allowing for easier visualization of the cochlear spiral, facilitating identification of 360°. If 360° can be identified, raters can easily add or subtract from that value (Fig. 5). A decrease in the number of skull X-RAYs used would decrease radiation and time, as well as lower hospital costs [19].

Rater experience may have a minor influence on the variability of skull X-RAY estimates. Although mean differences are similar, CI surgeons provided slightly lower standard deviation of paired differences than the other groups. However, their ratings were not much different from medical students when examining the Caldwell view alone (Table 3). This may be due to our low sample size for the specific skull X-RAY views. Due to the high accuracy of our rater estimates, this study brings into question the need for insertion depth estimates by radiologists. A post-hoc analysis of neuroradiologist insertion depth estimates on skull X-RAY demonstrated a mean of 343°, and a *p*-value of 0.79 when compared to CT. These values are highly comparable to our rater estimates.

Our results have important implications practically as skull X-RAYs provide many advantages compared to CTs. X-RAYs are less costly for institutions, easier to access, more comfortable for patients, and provide substantially less radiation. However, one disadvantage is the inability of skull X-RAYs to demonstrate intracompartemental placement.

There were a few limitations to this study. Firstly, only 13 patients with post-op CTs were included. At our centre, patients are typically given skull X-RAYs alone post-operatively to assess electrode insertion depth. In addition, CTs were only provided to patients who required a second contralateral CI, which may be a source of selection bias. There was also a delay between taking X-RAY and CT images for several patients of 1 to 2 years. During this time, the electrode may have migrated affecting the accuracy of direct comparisons [20]. Lastly, the Stenvers view was not routinely available for our patients and was not assessed. However, this view is commonly used in cochlear implantation studies to assess insertion depth [12, 21, 22].

Due to the recent influx of studies evaluating the impact of insertion depth, revision surgery for insertion depth may be an important area of future study. There is no indication in the literature that post-op imaging of CIs significantly alters management. Coombs et al. reported no immediate revisions after 220 CI cases [11]. However, in our experience revision surgery is usually required prior to activation if there were gross abnormalities in electrode placement (i.e. tip rollover, insertion into vestibule, significant electrode extrusion), and insertion depth has the potential to be included as well.

Further studies should include prospective trials to evaluate insertion depth, and also focus on determining optimal CI insertion depths to maximize hearing preservation. Pelliccia et al. suggested an insertion depth of 270° to optimize sound perception while minimizing cochlear trauma. Similarly, Nayak et al. suggested in the pediatric population, optimal hearing outcomes are observed with insertion depths of 270° to 360° [21, 23]. Once optimal insertion depths are determined, techniques to intra-operatively assess and change electrode depth will become important. This can be achieved by using landmarks on the array, which can then be monitored with post-operative X-RAY [23].

## Conclusions

Skull X-RAYs provide accurate measurements of CI insertion depth, supporting their use in the post-operative setting. In addition, the Caldwell view alone may be sufficient for evaluations of insertion depth, and experience has a minor impact on the variability of estimates.

## Additional file

Additional file 1: Data. (XLSX 622 kb)

## Abbreviations

CI: Cochlear Implant
CT: Computerized Topography
ICC: intra-class correlation
SHSC: Sunnybrook Health Sciences Centre
